# Effect of phosphodiesterase inhibitors on medication-related osteonecrosis of the jaw in mice: a comparison of phosphodiesterase-4 and phosphodiesterase-5 inhibition

**DOI:** 10.2340/aos.v84.45206

**Published:** 2025-12-29

**Authors:** Yunus Baş, Olgun Topal, Gökhan Sadi, Esra Aslan, Halit Buğra Koca, Mehmet Bilgehan Pektaş

**Affiliations:** a Private Dental Clinic, Afyonkarahisar, Türkiye; b Department of Oral and Maxillofacial Surgery, Faculty of Dentistry, Afyonkarahisar Health Sciences University, Afyonkarahisar, Türkiye; c Department of Biology, K.O. Science Faculty, Karamanoglu Mehmetbey University, Karaman, Türkiye; d Department of Histology and Embryology, Faculty of Medicine, Afyonkarahisar Health Sciences University, Afyonkarahisar, Türkiye; e Department of Medical Biochemistry, Faculty of Medicine, Afyonkarahisar Health Sciences University, Center, Afyonkarahisar, Türkiye; f Department of Medical Pharmacology, Faculty of Medicine, Afyonkarahisar Health Sciences University, Center, Afyonkarahisar, Türkiye

**Keywords:** Medication-related osteonecrosis of the jaw, zoledronic acid, sildenafil, rolipram, angiogenesis

## Abstract

**Objective:**

Medication-related osteonecrosis of the jaw lacks proven pharmacological prevention. We compared a phosphodiesterase-4 inhibitor rolipram and a phosphodiesterase-5 inhibitor sildenafil in a zoledronic acid– and dexamethasone–based mouse model with maxillary incisor extraction.

**Materials and Methods:**

Aged female mice were assigned to control, rolipram, or sildenafil after osteonecrosis induction and tooth extraction. Outcomes included histology of the extraction socket, immunostaining for vascular endothelial growth factor A (H-score), plasma cytokines, and bone-tissue gene expression measured by real-time quantitative polymerase chain reaction.

**Results:**

Circulating pro-inflammatory cytokines were lower with both inhibitors, whereas selected transcripts in bone were relatively higher, a pattern consistent with early, localized repair signaling during socket healing. Vascular endothelial growth factor A increased at transcript and protein levels. Both treatments were associated with higher blood-vessel and osteocyte counts; sildenafil additionally showed a clearer reduction in empty lacunae versus vehicle. Across inflammatory and angiogenic readouts, sildenafil produced more consistent improvements than rolipram.

**Conclusions:**

In this mouse model of medication-related osteonecrosis of the jaw, phosphodiesterase-5 inhibition outperformed phosphodiesterase-4 inhibition across histological and molecular outcomes. These findings suggest potential benefit and support further preclinical validation and hypothesis-generating clinical research.

## Introduction

Bisphosphonates are widely prescribed for the management of diseases characterized by excessive bone resorption, such as osteoporosis, bone metastases, and Paget’s disease [1]. By inhibiting the mevalonate pathway and suppressing osteoclast-mediated bone turnover, these drugs effectively strengthen bone but can also impair angiogenesis and compromise cellular viability within osseous tissue [2]. Long-term bisphosphonate therapy has been linked to the development of medication-related osteonecrosis of the jaw (MRONJ), a condition characterized by exposed necrotic bone in the maxillofacial region [3].

The precise pathogenesis of MRONJ remains incompletely understood; however, several risk factors – such as dental extraction, local infection, and reduced vascularization – are well recognized [4]. The 2022 position paper of the American Association of Oral and Maxillofacial Surgeons (AAOMS) emphasizes that MRONJ arises from a complex interaction of inflammation, impaired angiogenesis, and disrupted bone remodeling [5]. Current management strategies – mainly conservative approaches including antiseptic rinses, antibiotics, and limited surgical intervention – often fail to achieve full mucosal coverage or functional bone regeneration [6, 7]. Therefore, therapies that can both suppress inflammation and promote angiogenesis may offer improved outcomes in MRONJ management.

Experimental animal models have been fundamental in elucidating MRONJ mechanisms and evaluating potential interventions [8]. Zoledronic acid, a potent nitrogen-containing bisphosphonate, is the most widely used agent in preclinical MRONJ models due to its consistent ability to replicate the clinical and histological features of the disease [9, 10]. Despite considerable preclinical and clinical research, however, no pharmacological treatment has been firmly established to prevent or reverse MRONJ.

Recent studies have drawn attention to phosphodiesterase (PDE) inhibitors for their dual anti-inflammatory and proangiogenic effects [11]. Rolipram, a selective phosphodiesterase type 4 (PDE4) inhibitor, increases intracellular cyclic adenosine monophosphate (cAMP) levels, while sildenafil, a selective phosphodiesterase type 5 (PDE5) inhibitor, elevates cyclic guanosine monophosphate (cGMP) concentrations [12, 13]. Both cyclic nucleotides modulate vascular tone, endothelial function, and immune responses, influencing inflammation and tissue regeneration [14]. PDE5 inhibition, in particular, has demonstrated significant angiogenic and osteogenic benefits in ischemic and osteopenic models [15, 16].

Given these mechanisms, PDE inhibition may represent a promising therapeutic approach for MRONJ. This study was designed to compare the effects of PDE4 inhibition (rolipram) and PDE5 inhibition (sildenafil) in a murine model of zoledronic acid–induced osteonecrosis. We hypothesized that PDE5 inhibition would more effectively restore angiogenesis and support bone healing than PDE4 inhibition through enhanced nitric oxide–cGMP signaling. To test this, we analyzed molecular markers of inflammation and bone remodeling along with histological and immunohistochemical outcomes, providing insight into the comparative potential of these two pharmacological pathways.

## Materials and methods

### Animals

All experimental procedures were approved by the Afyon Kocatepe University Animal Research Ethics Committee (approval no. 49533702/102) and conducted in compliance with the ARRIVE 2.0 guidelines. A priori power analysis (α = 0.05, [1−β] = 0.80) determined that a minimum of 30 mice (10 per group) was sufficient to detect significant group differences.

Thirty 64-week-old female BALB/c mice (35–45 g) were obtained from the Pamukkale Experimental Animals Application and Research Center. Animals were housed under controlled conditions (22 ± 2°C, 12-h light/dark cycle) and fed a standard rodent diet (Korkutelim Yem Sanayi A.Ş., Antalya, Türkiye) with ad libitum access to food and water.

A separate healthy control group was not included because the primary objective was to compare the therapeutic effects of PDE inhibitors – rolipram and sildenafil – under standardized bisphosphonate-induced osteonecrosis conditions. Baseline data on healthy controls have been extensively characterized in previous MRONJ studies, allowing ethical reduction of animal use consistent with the 3Rs principle (Reduction, Replacement, Refinement) [8, 9].

### Experimental design and drug administration

All animals received zoledronic acid (Zometa®, 0.2 mg/kg; Novartis, USA) and dexamethasone (10 mg/kg; Mylan, USA) via intraperitoneal injection twice weekly for 8 weeks to induce MRONJ, following previously validated protocols [9, 10, 17]. Zoledronic acid was selected for its strong antiresorptive and antiangiogenic properties, while dexamethasone was co-administered to potentiate the osteonecrotic response by suppressing immune and vascular activity.

At week 8, animals were anesthetized with ketamine HCl (50 mg/kg; Ketalar®, Eczacıbaşı-Warner Lambert, Türkiye) and xylazine (10–20 mg/kg; Rompun 2%, Bayer, Türkiye). Local anesthesia (2% lidocaine HCl with 1:100,000 epinephrine) was applied prior to tooth extraction to minimize trauma and bleeding. The left maxillary central incisor was extracted under aseptic conditions using a periotome and micro-forceps, and a standardized alveolar defect (~0.5 mm) was created with a round bur at 1,500 rpm. All surgeries were performed by the same operator to ensure procedural consistency [18].

After MRONJ model induction, animals were randomly assigned to three groups (*n* = 10 per group):

Control group: zoledronic acid and dexamethasone only, no additional treatment.

Rolipram (Rol) group: zoledronic acid, dexamethasone, and rolipram.

Sildenafil (SDF) group: zoledronic acid, dexamethasone, and sildenafil.

The experimental groups and intervention schedule are summarized in Table 1.

**Table 1:** Experimental groups and interventions.

All mice received zoledronic acid (0.2 mg/kg, i.p.) and dexamethasone (10 mg/kg, i.p.) twice weekly for 8 weeks. The upper left maxillary central incisor was extracted at week 8. Post-extraction interventions were administered for 4 weeks. *n* = 24		
Control	Rol	SDF
*n* = 8	*n* = 8	*n* = 8
Vehicle (i.p. saline, 5×/week)	Rolipram (20 mg/kg, i.p., 5×/week)	Sildenafil (12 mg/kg, oral gavage, 5×/week)

i.p.: intraperitoneal; Rol: rolipram; SDF: sildenafil.

Note: Vehicle contained saline with 1.2% DMSO; dosing volumes were matched across groups.

Post-extraction treatments lasted for 4 weeks:

Rolipram (M02441, Glentham Life Sciences, Germany) was administered intraperitoneally at 20 mg/kg, five times per week, consistent with its effective anti-inflammatory and osteogenic dose range [19].

Sildenafil citrate (GP3242, Glentham Life Sciences, Germany) was administered orally by gavage at 12 mg/kg, five times per week, as described in previous studies demonstrating bone regeneration and angiogenic activity [20].

Both drugs were dissolved in isotonic saline containing 1.2% dimethyl sulfoxide (DMSO). At the end of treatment, mice were euthanized with a ketamine (100 mg/kg) and xylazine (10 mg/kg) overdose. Maxillary bones were harvested – half fixed in 10% neutral-buffered formalin for histology, half stored at −85°C for molecular analyses. Blood samples were collected via cardiac puncture, centrifuged (10,000 × g, 10 min, 4°C), and plasma stored at −85°C for cytokine analysis.

### Histological and immunohistochemical evaluation

Fixed maxillary tissues were decalcified, paraffin-embedded, and sectioned at 5 µm. Sections were stained with hematoxylin–eosin (H&E) to assess bone microarchitecture, including osteocyte density, vascularization, and empty lacunae.

For vascular endothelial growth factor A (VEGFA) detection, antigen retrieval was performed in citrate buffer (pH 6.0), endogenous peroxidase activity was quenched with 3% hydrogen peroxide, and slides were incubated overnight at 4°C with anti-VEGFA antibody (sc-7269, Santa Cruz Biotechnology; 1:100). Detection was performed using horseradish peroxidase (HRP)-conjugated secondary antibody (Thermo Scientific, USA) and 3-amino-9-ethylcarbazole (AEC) chromogen, followed by counterstaining with Mayer’s hematoxylin.

Immunostaining intensity was semi-quantitatively evaluated using the H-score formula:

H = Σ (I + 1) × PC,

where I represents staining intensity (0 = none, 1 = weak, 2 = moderate, 3 = strong) and PC represents the percentage of cells stained at each intensity. A minimum of 100 cells per sample were scored at 400× magnification by two blinded observers.

### Quantitative real-time PCR

Total RNA was extracted from ~50 mg of frozen maxillary bone using the RNeasy Mini Kit (Qiagen, Venlo, Netherlands). RNA purity was verified spectrophotometrically (A260/A280 ratio), and integrity confirmed by agarose gel electrophoresis. cDNA was synthesized from 500 ng RNA using oligo(dT)18 primers and a First-Strand cDNA Synthesis Kit (MBI Fermentas, USA). Primer sequences used for RT-qPCR are provided in Table 2.

**Table 2:** Primer sequences for IL-1β, IL-6, TGF-α, TGF-β, TRAP, CTSK, NFATc1, VEGFA, OCN, OPN, OPG, RANK, and GAPDH used for RT-qPCR.

Gene	Forward primer sequence (5′→3′)	Reverse primer sequence (3′→5′)
IL-1β	GCCACCTTTTGACAGTGATG	CACGGGAAAGACACAGGTAG
IL-6	GTCCTTCCTACCCCAATTTCC	TGCCGAGTAGATCTCAAAGTG
TGFα	TAGGTTGAGACAAGTCCGGT	TCAACTGTACTGCCCTCTGA
TGFβ	GATGTTCTCTCGTTCTGTCTCA	AGACCATAAATTGCAGGTTGC
TRAP	ACCAGCAAGGATTGCGAGGCAT	GGATGACAGACGGTATCAGTGG
CTSK	GGATGAAATCTCTCGGCGTT	GTCCCGTCATCTTCTGAACC
NFATc1	AGTGAGCTGAGAGGAGGTTT	AGATATACACCCCCAGACCG
VEGFA	CTGCTGTAACGATGAAGCCCTG	GCTGTAGGAAGCTCATCTCTCC
OCN	GCAATAAGGTAGTGAACAGACTCC	CCATAGATGCGTTTGTAGGCGG
OPN	TGACGATGATGATGACGATGG	TGGCTATAGGATCTGGGTGC
OPG	CAATGGCTGGCTTGGTTTCATAG	CTGAACCAGACATGACAGCTGGA
RANK	CATCCCTTGCAGCTCAACAA	GAGCAGAACGATGAGACTGG
GAPDH	TAACATCAAATGGGGTGAGG	GGTTCACACCCATCACAAAC

Quantitative real-time polymerase chain reaction (RT-qPCR) was performed using SYBR Green Master Mix (Roche, Switzerland) on a LightCycler 480 II system. Cycling parameters were as follows: 95°C for 10 min, followed by 45 cycles of 95°C for 10 s, 58°C for 15 s, and 72°C for 15 s. Specificity was confirmed by melt-curve analysis and the absence of amplification in no-template controls.

Gene expression levels of IL-1β, IL-6, TGFα, TGFβ, TRAP, CTSK, NFATc1, VEGFA, OCN, OPN, OPG, and RANK were normalized to glyceraldehyde-3-phosphate dehydrogenase (GAPDH) using the ΔΔCt method [16]. All reactions were run in triplicate.

### Plasma cytokine quantification (ELISA)

Plasma concentrations of TNF-α, IL-1β, IL-17, and IL-23 were measured using commercial enzyme-linked immunosorbent assay (ELISA) kits (BT Lab Bioassay Technology Laboratory, Zhejiang, China). Samples and standards were assayed in duplicate, and absorbance was read at 450 nm using a ChemWell 2910 microplate reader (Awareness Technology, Palm City, USA). Cytokine concentrations were interpolated from standard curves and expressed as pg/mL.

### Statistical analysis

Each mouse was treated as an independent biological replicate (*n* = 8–10 per group). Technical replicates from RT-qPCR (triplicates) and ELISA (duplicates) were averaged for each sample prior to statistical testing.

Normality of residuals was verified using the Shapiro–Wilk test and visual inspection of Q–Q plots. Homogeneity of variances was tested using Levene’s test. If assumptions were met, one-way analysis of variance (ANOVA) followed by Tukey’s honestly significant difference (HSD) post hoc test was applied. When assumptions were violated, data were analyzed using the Kruskal–Wallis test with Dunn–Holm correction for multiple comparisons.

Data are reported as mean ± standard deviation (SD) for normally distributed variables and median (interquartile range) for nonparametric data. Exact *p*-values are reported unless *p* < 0.0001. Two-tailed significance was defined as *p* < 0.05. All analyses were conducted using IBM SPSS Statistics v29.0.2.0 (IBM Corp., Armonk, NY, USA) and GraphPad Prism v10.0 (GraphPad Software, San Diego, CA, USA).

## Results

All animals were examined intraorally every two days to determine whether there was any irritation in the tongue and lips; their weights, as well as their food and liquid intake were assessed. No major findings were encountered during the experimental period that would affect the study results.

### Effects of Rol and SDF on the mRNA expression of IL-1β, IL-6, transforming growth factor α and β, tartrate-resistant acid phosphatase, cathepsin K, activated T-cell cytoplasmic factor 1, VEGFA, osteocalcin, osteopontin, osteoprotegerin, and RANK in the maxillary bone of mice

**Figure 1:**
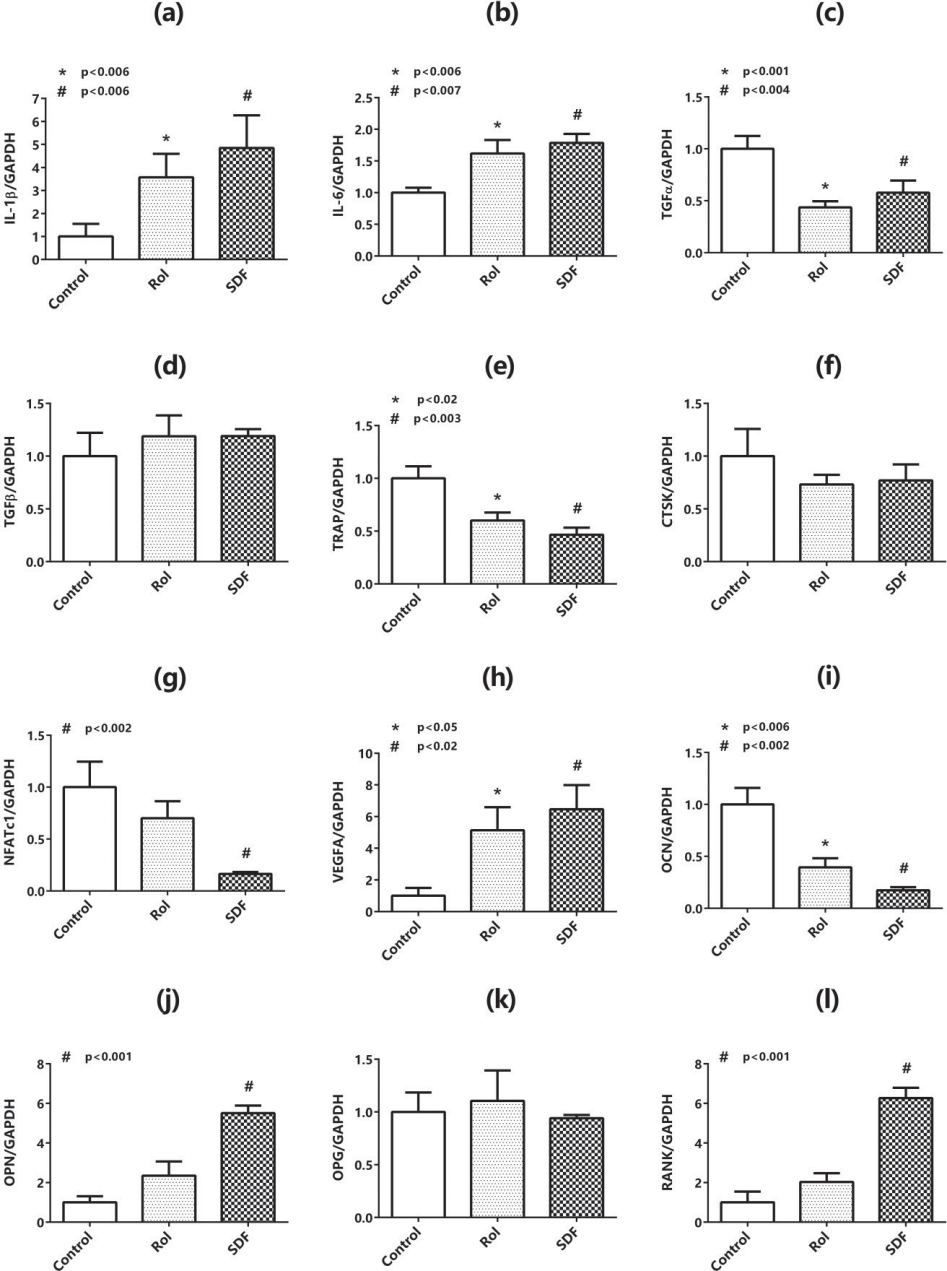
Bone-tissue mRNA expression (RT-qPCR). Relative expression (fold-change vs. vehicle; mean ± SD, *n* = 8/group) for IL-1β (a), IL-6 (b), TGF-α (c), TGF-β (d), TRAP (e), CTSK (f), NFATc1 (g), VEGFA (h), OCN (i), OPN (j), OPG (k), RANK (l) in maxillary bone. Omnibus and pairwise exact *p*-values (ANOVA/Tukey or Kruskal–Wallis/Dunn–Holm) are displayed on each panel. Symbols: * indicates rolipram versus vehicle; # indicates sildenafil versus vehicle; † indicates sildenafil versus rolipram. Primer sequences and cycling conditions are provided in Table 2.

Maxillary bone samples were isolated from the mice, and the expression of IL-1β (Figure 1a), IL-6 (Figure 1b), transforming growth factor α (TGFα) (Figure 1c), TGFβ (Figure 1d), tartrate-resistant acid phosphatase (TRAP) (Figure 1e), cathepsin K (CTSK) (Figure 1f), activated T-cell cytoplasmic factor 1 (NFATc1) (Figure 1g), VEGFA (Figure 1h), osteocalcin (OCN) (Figure 1i), osteopontin (OPN) (Figure 1j), osteoprotegerin (OPG) (Figure 1k), and RANK (Figure 1l) was measured by real-time PCR. Maxillary bone IL-1β, IL-6, VEGFA, OPN, and RANK mRNA expression was significantly enhanced in the SDF group compared with the control, whereas TGFα, TRAP, NFATc1, and OCN were decreased. No changes were observed in TGFβ, CTSK, and OPG mRNA expression. Similarly, given that Rol increased IL-1β, IL-6, and VEGFA mRNA expression compared with the control, OPN and RANK levels tended to increase insignificantly. However, TGFα, TRAP, and OCN expression decreased, and no changes were observed in the expression of TGFβ, CTSK, and OPG mRNA. Remarkably, CTSK expression tended to decrease in both Rol and SDF groups, but this change was not significant.

### Effects of Rol and SDF on the TNFα, IL-1β, IL-17, and IL-23 expression

**Figure 2:**
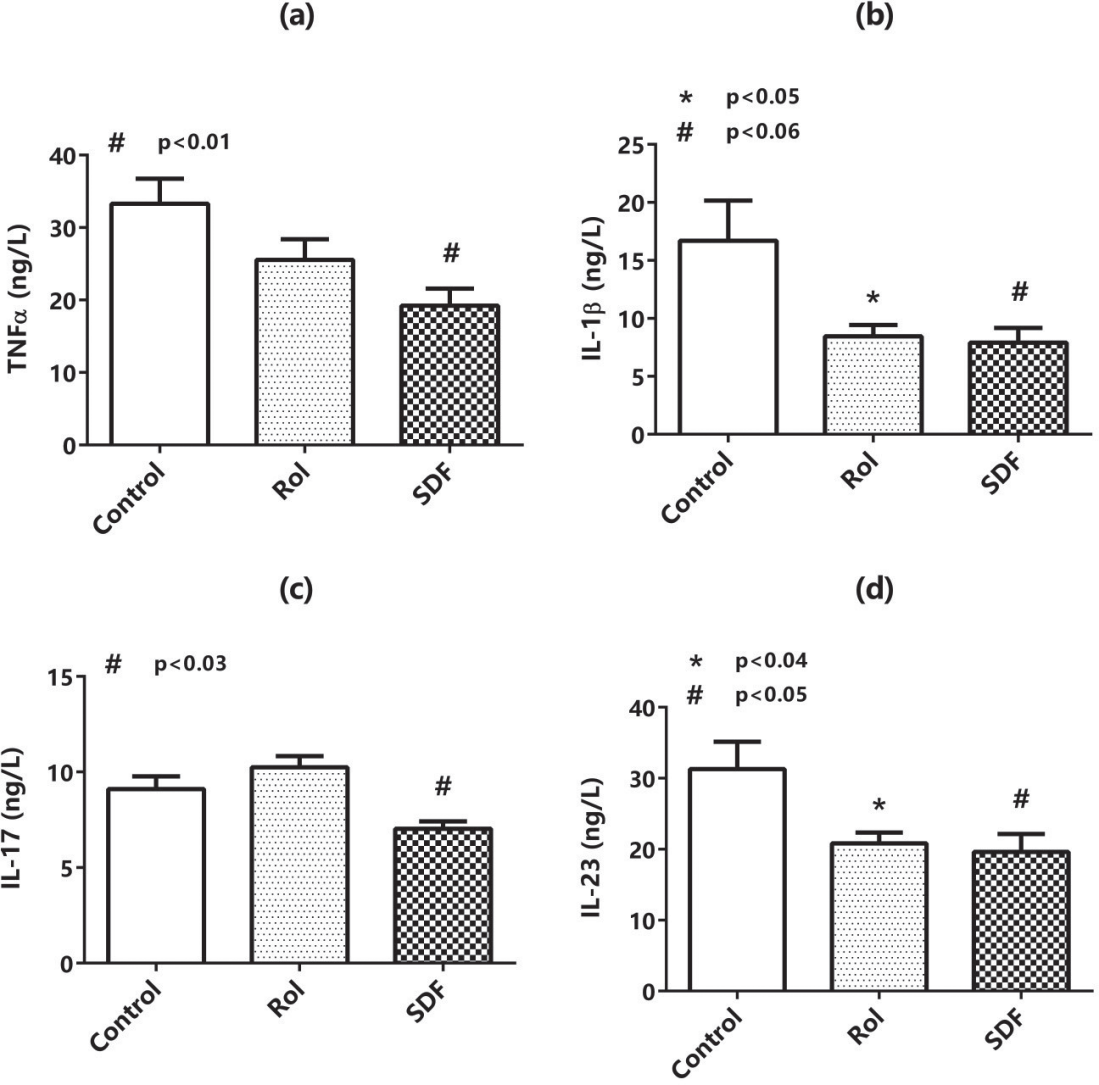
Plasma cytokines (ELISA). Concentrations (pg/mL; mean ± SD, *n* = 8/group) of TNF-α (a), IL-1β (b), IL-17 (c), IL-23 (d). Omnibus and pairwise exact *p*-values (ANOVA/Tukey or Kruskal–Wallis/Dunn–Holm) are shown on the panels. Symbols: * rolipram versus vehicle; # sildenafil versus vehicle; † sildenafil versus rolipram.

TNFα, IL-1β, IL-17, and IL-23 expression in the plasma samples of mice was measured using ELISA kits (Figure 2a–d). The results revealed that SDF significantly decreased TNFα, IL-1β, IL-17, and IL-23 levels compared with the control. However, Rol reduced IL-1β and IL-23 levels, whereas TNFα expression tended to decrease, but this change was not significant. However, Rol did not change IL-17 protein expression.

### Effects of Rol and SDF on VEGFA expression and histological parameters

**Figure 3:**
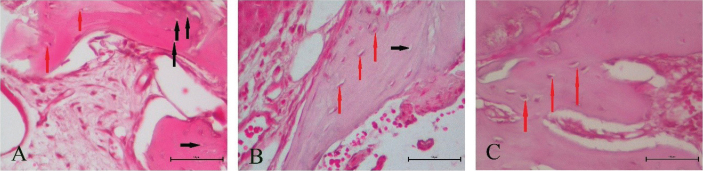
Representative histology of extraction sockets. Hematoxylin–eosin–stained transverse sections of maxillary bone at the extraction site (×400; scale bar 50 µm) from vehicle, rolipram, and sildenafil groups. Black arrows denote empty osteocyte lacunae; red arrows denote viable osteocytes.

**Figure 4:**
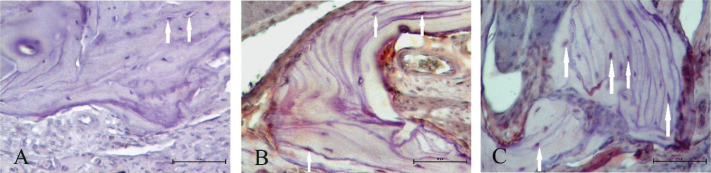
VEGFA immunohistochemistry. Representative immunostaining of VEGFA in maxillary bone (×400; scale bar 50 µm). White arrows indicate VEGFA-positive cells. Both the number of stained cells and staining intensity are visually lower in vehicle compared with treatment groups.

**Figure 5:**
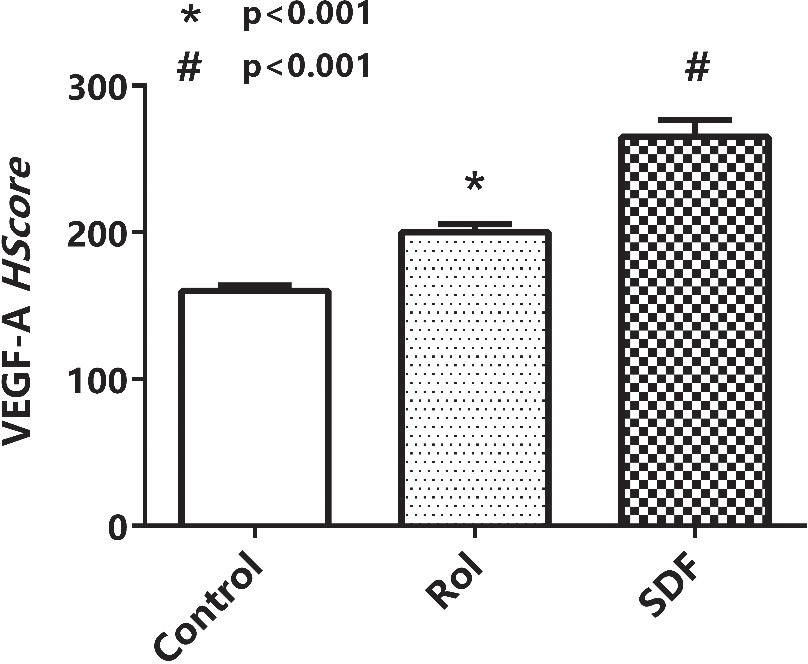
VEGFA immunoreactivity (H-score). Semi-quantitative H-scores (0–300; mean ± SEM, *n* = 6–8 per group) for VEGFA immunostaining. * indicates rolipram versus vehicle *p* < 0.05; # indicates sildenafil versus vehicle *p* < 0.05.

HScore results of VEGFA immunostaining indicated that Rol and SDF triggered VEGFA expression compared with the control (Figure 3). The increase is shown in Figure 4. Blood vessels, osteocytes, and empty lacunae are presented in Figure 5. According to the histological results, Rol and SDF significantly increased the number of blood vessels and osteocytes compared with control. By contrast, SDF significantly reduced the number of empty lacunae; a tendency toward decrease was observed with Rol, but it was not significant. Quantitative histological findings are summarized in Table 3.

**Table 3:** Quantitative histology.

Groups	Blood Vessels	*p*	Osteocytes	*p*	Empty Lacunae	*p*
Control	7 ± 0.4	-	54 ± 4.5	-	23.8 ± 1.2	-
Rol	12.2 ± 1.1*	0.0002	73.2 ± 2*	0.0008	19.5 ± 2.5	0.1353
SDF	17.5 ± 1.8#	< 0.0001	101 ± 7.1#	< 0.0001	11.2 ± 1.9	0.0941

Values are mean ± SD counts per 20× field for blood vessels, viable osteocytes, and empty osteocyte lacunae by group (n = 8/group). Exact p-values are shown for pairwise comparisons versus vehicle (Control) (and versus rolipram where applicable).

* p < 0.05, rolipram versus vehicle (Control).

# p < 0.05, sildenafil versus vehicle (Control). Field selection and counting procedures are described in the Methods.

## Discussion

Bisphosphonates are effective agents for the management of osteoporosis and cancer-related bone disease owing to their ability to inhibit osteoclastic bone resorption and angiogenesis. However, prolonged use is associated with MRONJ, a condition characterized by exposed necrotic bone, inflammation, and impaired vascularization [21]. Epidemiological studies indicate that MRONJ is more prevalent in older women, particularly after menopause [22]. To model this risk group, aged female mice were selected, corresponding to the postmenopausal period in humans [23].

Zoledronic acid, a potent nitrogen-containing bisphosphonate, was chosen for MRONJ induction due to its high osteoclast-inhibitory activity and strong antiangiogenic potential [17]. The co-administration of dexamethasone amplifies inflammatory and metabolic disturbances, facilitating reproducible osteonecrotic lesions [24]. Although MRONJ occurs more frequently in the mandible, the extraction of maxillary incisors in rodents is generally preferred to minimize surgical mortality and ensure procedural consistency [18].

PDE inhibitors have gained attention as potential therapeutic agents for bone regeneration because they modulate both inflammation and angiogenesis [25, 26]. Rolipram, a PDE4 inhibitor, acts by elevating intracellular cAMP levels, suppressing inflammatory cytokine production [19, 27]. Sildenafil, a PDE5 inhibitor, increases cGMP, thereby promoting nitric oxide–dependent vasodilation and endothelial repair [20, 28]. These distinct mechanisms suggest that PDE inhibition could mitigate MRONJ pathology by re-establishing vascular supply and reducing chronic inflammation.

In this study, both rolipram and sildenafil improved socket healing and bone architecture. Histological analysis demonstrated a significant increase in osteocyte number and vascular density, coupled with a reduction in empty lacunae, particularly in the sildenafil group. Immunohistochemical staining revealed elevated VEGFA expression, supporting the hypothesis that enhanced angiogenesis contributes to improved tissue repair. Similar proangiogenic effects of PDE inhibition have been previously documented in experimental bone and ischemic models [28, 29].

At the molecular level, sildenafil markedly upregulated VEGFA, OPN, RANK, IL-1β, and IL-6 mRNA expression, while reducing TRAP, NFATc1, and TGFα, which are linked to osteoclastic activity. These changes suggest a shift toward osteoblastic and angiogenic pathways that favor bone remodeling. Rolipram showed similar but less pronounced effects, indicating that cGMP-mediated signaling exerts stronger regulatory influence on bone metabolism than cAMP alone [19, 27]. The concurrent decrease in proresorptive genes such as TRAP and NFATc1 suggests a suppression of osteoclast differentiation, aligning with previous findings on PDE-mediated modulation of osteoimmune responses [30].

Systemic cytokine analysis confirmed that both treatments attenuated inflammation, with sildenafil producing significant reductions in TNFα, IL-1β, IL-17, and IL-23 plasma levels. These cytokines are known to exacerbate osteoclastic activity through RANKL upregulation [30]. The paradoxical increase in IL-1β and IL-6 mRNA in local bone tissue may represent a controlled reparative immune activation essential for bone remodeling rather than persistent inflammation.

Collectively, these findings suggest that PDE inhibition, particularly via the PDE5 pathway, may promote bone regeneration by restoring angiogenic balance and attenuating proinflammatory signaling. The more pronounced response observed with sildenafil likely reflects the dual action of cGMP elevation: stimulation of VEGFA-mediated vascularization and enhancement of osteogenic differentiation.

This study has several limitations. Firstly, a healthy control group was not included because the primary aim was to compare the relative therapeutic effects of PDE4 and PDE5 inhibition under standardized MRONJ conditions. The omission reduced animal use in accordance with ethical guidelines, but limits interpretation relative to normal bone healing. Secondly, the experimental duration was relatively short, precluding the assessment of late-stage bone remodeling and long-term tissue stability. Thirdly, only female mice were used to model the postmenopausal population, restricting extrapolation to male physiology. Future investigations including both sexes, extended observation periods, and mechanistic analyses of signaling pathways are warranted to confirm these results.

## Conclusion

In this murine model of bisphosphonate-induced osteonecrosis, both PDE4 and PDE5 inhibition improved bone healing by enhancing angiogenesis and reducing inflammatory cytokine activity. Sildenafil demonstrated more consistent effects across histological, biochemical, and molecular parameters. These results suggest that PDE5 inhibition may provide a promising pharmacologic approach for managing or preventing MRONJ. However, given the preclinical nature of this study, translation to clinical settings should be approached with caution. Further research is required to clarify underlying mechanisms, optimize dosing, and confirm efficacy in larger animal and clinical models.

## References

[1] Russell RGG, Watts NB, Ebetino FH, Rogers MJ. Mechanisms of action of bisphosphonates: similarities and differences and their potential influence on clinical efficacy. Osteoporos Int. 2008;19(6):733–59. 10.1007/s00198-007-0540-8 18214569

[2] Santini D, Schiavon G, Angeletti S, Vincenzi B, Gasparro S, Grilli C, et al. Last generation of amino-bisphosphonates (N-BPs) and cancer angiogenesis: a new role for these drugs? Recent Pat Anticancer Drug Discov. 2006;1(3):383–96. 10.2174/157489206778776989 18221048

[3] Ruggiero SL, Dodson TB, Assael LA, Landesberg R, Marx RE, Mehrotra B. Medication-related osteonecrosis of the jaw: definition and best practice recommendations. J Oral Maxillofac Surg. 2014;72(10):1938–56. 10.1016/j.joms.2014.04.031 25234529

[4] Yoneda T, Hagino H, Sugimoto T, Oshima Y, Nakashima T, Hiraga T, et al. Antiresorptive agent-related osteonecrosis of the jaw: position paper 2023. Bone Rep. 2023;18:101652.

[5] American Association of Oral and Maxillofacial Surgeons (AAOMS). Position paper on medication-related osteonecrosis of the jaw – 2022 update. J Oral Maxillofac Surg. 2022;80(5):920–43. 10.1016/j.joms.2022.02.008 35300956

[6] Varoni EM, Lombardi N, Villa G, Pispero A, Sardella A, Lodi G. Conservative management of medication-related osteonecrosis of the jaws (MRONJ): a retrospective cohort study. Antibiotics (Basel). 2021;10(2):195. 10.3390/antibiotics10020195 33671429 PMC7922963

[7] Otto S, Pautke C, Van den Wyngaert T, Niepel D, Schiødt M. Medication-related osteonecrosis of the jaw: prevention, diagnosis and management in patients with cancer and bone metastases. Cancer Treat Rev. 2018;69:177–187. 10.1016/j.ctrv.2018.06.007 30055439

[8] Aguirre JI, Castillo EJ, Kimmel DB. Preclinical models of medication-related osteonecrosis of the jaw (MRONJ). Bone. 2021;153:116184. 10.1016/j.bone.2021.116184 34520898 PMC8743993

[9] Li JW, Wang JY, Yu RQ, Huo L, Zheng LW. Expression of angiogenic markers in jawbones and femur in a rat model treated with zoledronic acid. BMC Res Notes. 2022;15(1):12. 10.1186/s13104-021-05900-5 35012647 PMC8751108

[10] Kim JW, Tatad JCI, Landayan MEA, Kim SJ, Kim MR. Animal model for medication-related osteonecrosis of the jaw with precedent metabolic bone disease. Bone. 2015;81:442–448. 10.1016/j.bone.2015.08.012 26297440

[11] Conti M, Beavo J. Biochemistry and physiology of cyclic nucleotide phosphodiesterases: essential components in cyclic nucleotide signaling. Annu Rev Biochem. 2007;76:481–511. 10.1146/annurev.biochem.76.060305.150444 17376027

[12] Zhang HT. Phosphodiesterase-4 inhibitors as potential antidepressants. Curr Pharm Des. 2009;15(14):1688–98. 10.2174/138161209788168092 19442182

[13] Boolell M, Allen MJ, Ballard SA, Gepi-Attee S, Muirhead GJ, Naylor AM, et al. Sildenafil: an orally active type 5 cyclic GMP-specific phosphodiesterase inhibitor for the treatment of penile erectile dysfunction. Int J Impot Res. 1996;8(2):47–52. 8858389

[14] Francis SH, Blount MA, Corbin JD. Mammalian cyclic nucleotide phosphodiesterases: molecular mechanisms and physiological functions. Physiol Rev. 2011;91(2):651–90. 10.1152/physrev.00030.2010 21527734

[15] Roy S, Kloner RA, Salloum FN, Jovin IS. Cardiac effects of phosphodiesterase-5 inhibitors: efficacy and safety. Cardiovasc Drugs Ther. 2023;37(4):793–806. 10.1007/s10557-021-07275-y 34652581 PMC9010479

[16] Dincel YM, Alagoz E, Arikan Y, Aydin M, Yildirim AO, Kose O, et al. Biomechanical, histological, and radiological effects of different phosphodiesterase inhibitors on femoral fracture healing in rats. J Orthop Surg (Hong Kong). 2018;26(2):2309499018777885. 10.1177/2309499018777885 29848169

[17] Dhillon S. Zoledronic acid (Reclast®, Aclasta®): a review in osteoporosis. Drugs. 2016;76(15):1683–97. 10.1007/s40265-016-0662-4 27864686

[18] Hadad H, Matheus HR, Pai SI, Souza FA, Guastaldi FPS. Rodents as an animal model for studying tooth extraction–related medication-related osteonecrosis of the jaw: assessment of outcomes. Arch Oral Biol. 2024;159:105875. 10.1016/j.archoralbio.2023.105875 38160519 PMC11729500

[19] Tokuhara Y, Wakitani S, Imai Y, Nomura C, Hoshino M, Yano K, et al. Local delivery of rolipram, a phosphodiesterase-4-specific inhibitor, augments bone morphogenetic protein-induced bone formation. J Bone Miner Metab. 2010;28(1):17–24. 10.1007/s00774-009-0103-5 19554392

[20] Pal S, Rashid M, Singh SK, Porwal K, Singh P, Mohamed R, et al. Skeletal restoration by phosphodiesterase 5 inhibitors in osteopenic mice: evidence of osteoanabolic and osteoangiogenic effects of the drugs. Bone. 2020;135:115305. 10.1016/j.bone.2020.115305 32126313

[21] Rogers SN, Palmer NOA, Lowe D, Randall C. United Kingdom nationwide study of avascular necrosis of the jaws including bisphosphonate-related necrosis. Br J Oral Maxillofac Surg. 2015;53(2):176–82. 10.1016/j.bjoms.2014.11.008 25497376

[22] Pietschmann P, Rauner M, Sipos W, Kerschan-Schindl K. Osteoporosis: an age-related and gender-specific disease – a mini-review. Gerontology. 2009;55(1):3–12. 10.1159/000166209 18948685

[23] Biguetti CC, De Oliva AH, Healy K, Mahmoud RH, Custódio IDC, Constantino DH, et al. Medication-related osteonecrosis of the jaws after tooth extraction in senescent female mice treated with zoledronic acid: microtomographic, histological and immunohistochemical characterization. PLoS One. 2019;14(6):e0214173. 10.1371/journal.pone.0214173 31199812 PMC6568384

[24] Barba-Recreo P, Del Castillo Pardo de Vera JL, García-Arranz M, Yébenes L, Burgueño M. Zoledronic acid–related osteonecrosis of the jaws: experimental model with dental extractions in rats. J Craniomaxillofac Surg. 2014;42(6):744–50. 10.1016/j.jcms.2013.11.005 24342733

[25] Yalcin-Ulker GM, Cumbul A, Duygu-Capar G, Uslu Ü, Sencift K. Preventive effect of phosphodiesterase inhibitor pentoxifylline against medication-related osteonecrosis of the jaw: an animal study. J Oral Maxillofac Surg. 2017;75(11):2354–68. 10.1016/j.joms.2017.04.017 28529150

[26] Delfrate G, Mroczek T, Mecca LEA, Andreis JD, Fernandes D, Lipinski LC, et al. Effect of pentoxifylline and α-tocopherol on medication-related osteonecrosis of the jaw in rats: before and after dental extraction. Arch Oral Biol. 2022;137:105397. 10.1016/j.archoralbio.2022.105397 35286947

[27] Schick MA, Schlegel N. Clinical implication of phosphodiesterase-4 inhibition. Int J Mol Sci. 2022;23(3):1209. 10.3390/ijms23031209 35163131 PMC8835523

[28] Ölmestig JNE, Marlet IR, Hainsworth AH, Kruuse C. Phosphodiesterase 5 inhibition as a therapeutic target for ischemic stroke: a systematic review of preclinical studies. Cell Signal. 2017;38:39–48. 10.1016/j.cellsig.2017.06.015 28648945

[29] de Campos Pessoa AL, de Oliveira Araújo VHV, Rosa Nascimento AL, Elias N, de Carvalho JJ. Phosphodiesterase-5 inhibition improves bone regeneration at the early stages of ischemic osteonecrosis of the femoral head in rats. J Orthop Res. 2021;39(10):2077–82. 10.1002/jor.24934 33270292

[30] Lombard T, Neirinckx V, Rogister B, Gilon Y, Wislet S. Medication-related osteonecrosis of the jaw: new insights into molecular mechanisms and cellular therapeutic approaches. Stem Cells Int. 2016;2016:8768162. 10.1155/2016/8768162 27721837 PMC5046039

